# Multiple factors in the prediction of risk of recurrent vitreous haemorrhage after sutureless vitrectomy for non-clearing vitreous haemorrhage in patients with diabetic retinopathy

**DOI:** 10.1186/s12886-020-01532-8

**Published:** 2020-07-16

**Authors:** Yuhua Ding, Bangtao Yao, Hui Hang, Hui Ye

**Affiliations:** 1 Department of Ophthalmology, Jiangsu Province Hospital, The First Affiliated Hospital of Nanjing Medical University, Nanjing, Jiangsu Province China; 2 Department of Ophthalmology, Lishui District People’s Hospital, Lishui branch of Southeast University Affiliated Zhongda Hospital, Nanjing, Jiangsu Province China

**Keywords:** Recurrent vitreous haemorrhage, Diabetic retinopathy, 23-gauge, 25-gauge, Age, Duration of diabetes, Creatinine

## Abstract

**Background:**

We aimed to analyse multiple factors in the prediction of risk of postoperative recurrent vitreous haemorrhage (RVH) for non-clearing vitreous haemorrhage in patients with diabetic retinopathy (DR) who underwent sutureless vitrectomy with 23- (23G) or 25-gauge (25G) narrow-gauge systems.

**Methods:**

A retrospective consecutive case series design was used. DR patients who underwent sutureless vitrectomy for non-clearing vitreous haemorrhage between June 2017 and October 2019 were enrolled. All operations were performed at a tertiary hospital. Patient demographics and risk factors, including age, gender, duration of diabetes, preoperative fasting blood sugar levels (FBSL), systolic blood pressure (SBP), serum creatinine (Cr), urea, triamcinolone acetonide (TA), electrical coagulation, air-fluid exchange, pan-retinal photocoagulation status (PRP), anti-vascular endothelial growth factor drug (anti-VEGF), and other factors, were recorded. Patients were divided into two groups based on the timing of their postoperative RVH: immediate postoperative RVH (within 2 weeks after operation) and delayed postoperative RVH (beyond 2 weeks after operation).

**Results:**

Overall, 167 eyes (167patients) were enrolled. Seventy eyes were underwent 23G and 25G sutureless vitrectomy performed in 97 eyes, respectively. Postoperative RVH developed in 18 eyes (25.7%) in Group 23G and in 20 eyes (21.6%) in Group 25G (*P* = 0.540). Of these, 3 eyes (4.3%) had severed RVH in Group 23G compared with 5 eyes (5.2%) in Group 25G (*P* = 0.584). Delayed postoperative RVH occurred in 6 eyes (8.6%) in Group 23G and 8 eyes (8.2%) in Group 25G (*P* = 0.789). A binomial logistic regression analysis revealed that age, duration of diabetes, and Cr level were significantly associated with RVH in both Group 23G (*P* < 0.05) and Group 25G (P < 0.05).

**Conclusions:**

The incidence and severity of RVH were 25.7 and 4.3%, respectively, in Group 23G and 21.6 and 5.2%, respectively, in Group 25G. Thus, the 23G sutureless vitrectomy approach was as safe as the 25G sutureless vitrectomy approach for treating vitreous haemorrhage in patients with DR. A younger age, shorter duration of diabetes, and higher Cr levels were risk factors for postoperative RVH in sutureless vitrectomy.

## Background

Diabetes mellitus (DM) is a common chronic metabolic disease with an increasing prevalence worldwide—more than half a billion individuals are projected to have DM by 2030 [1, 2]. Furthermore, diabetic retinopathy (DR) is the most common microvascular complication of DM and is responsible for up to 4.8% of blindness globally [3]. Despite the use of anti-vascular endothelial growth factor (anti-VEGF) drugs or laser for the treatment of diabetic macular oedema and proliferative DR (PDR) [4–6], vitrectomy surgery remains necessary in up to one-third of eyes with DR [7]. Vitreous haemorrhage (VH) is one of the most common PDR complications and the main cause for sudden vision loss in PDR patients [8]. Non-clearing VH (NCVH) is a good indicator of pars plana vitrectomy (PPV), with vision improvement reported in approximately 75% of PDR patients after diabetic vitrectomy [9]. However, one of the most common postoperative complications associated with NCVH is recurrent vitreous haemorrhage (RVH), which may cause visual impairment and require re-operation [10].

The incidence rate of RVH in PDR patients has been reported to range from 11.8 to 75% [11, 12]. Early studies have found that the most common causes of RVH after vitrectomy are fibrovascular ingrowth at the sclerotomy sites, residual or recurrent neovascular membrane formation on the retina, and insufficient retinal photocoagulation [13, 14].

In the present study, we focused on a series of patients with DR who were undergoing sutureless 23G or 25G vitrectomy for NCVH. We aimed to analyze the incidence and possible risk factors for RVH following sutureless 23G and 25G vitrectomies.

## Methods

A retrospective consecutive case series design was used. Patients with DR (both type I diabetes and type II diabetes) undergoing sutureless vitrectomies for NCVH between June 2017 and October 2019 at a tertiary hospital (The First Affiliated Hospital of Nanjing Medical University, Jiangsu Province Hospital) were enrolled. VH cases included cases associated with TRD and fibrovascular membranes, not only simple VH cases. We excluded cases with a history of previous scleral buckling, PPV, other coexisting ocular disorders such as glaucoma and uveitis, use of sutures for the incisions, silicone oil or inert gas injection during the operation.

A variety of pre-, intra-, and postoperative patient characteristics were assessed. Characteristics included patient age, gender, duration of DM, visual acuity, intraocular pressure (IOP), anterior and posterior segment findings, a history of pan-retinal photocoagulation (PRP), the date of surgery, and any subsequent visits were recorded. Preoperative test results such as fasting blood sugar levels (FBSL), systolic blood pressure (SBP), serum creatinine (Cr), blood urea, prothrombin time (PT), activated partial thromboplastin time (APTT), and prothrombin standardisation ratio (PT-INR) were also recorded. Surgical characteristics including usage of triamcinolone acetonide (TA), electrical coagulation, air-fluid exchange, pre-PRP, and anti-VEGF use were determined from the patient’s operative record. All patients were followed-up routinely at 1 day, 1 week, 2 weeks, 4 weeks, 2 months, 3 months, and 6 months post-operation. Depending on the commencement of RVH, patients were divided into immediate postoperative RVH (within 2 weeks after operation) and delayed postoperative RVH (beyond 2 weeks after operation) groups. In addition, the severity of postoperative RVH was classified as follows: grade 0 (no haemorrhage), grade 1 (mild-to-moderate: optic disc, macula, and retinal vessel visibility), or grade 2 (severe: retina details were not seen).

All operations were performed by subspecialist vitreoretinal consultants who had more than 10 years of experience. All operations were performed under peribulbar anaesthesia and using the Alcon Constellation 23G or 25G systems (Alcon, Geneva, Switzerland). A wide-angle non-contact indirect viewing system with a built-in image inverter was used in all cases. All patients underwent standard three-port PPV with a combination of dissection and removal of the fibrovascular membranes and vitreoretinal traction. TA staining, electrical coagulation for the remnant of fibrovascular membranes during the operation, air-fluid exchange, and extensive laser photocoagulation surgery were performed as required. No intraocular tamponade (silicone oil or inert gas) was used. At the end of the procedure, cannulas were carefully removed without suturing. Phacoemulsification surgery was performed to remove the clouded lens, which affected the vitrectomy, and an intraocular len was implanted when required. Preoperative anti-VEGF therapy was used in selected patients with informed consent given their extent of neovascularisation. This study followed the tenets of the Declaration of Helsinki. Approval from the appropriate ethics committee was obtained, and informed consent was acquired from all patients before surgery.

### Statistical analyses

All analyses were performed using SPSS version 23.0 for Windows. Descriptive statistics were determined. To compare the two groups, univariate analyses using Chi-squared test, Fisher’s test and T-test were performed, as appropriate. Binomial logistic regression analyses and multivariate analysis were also performed. A two-sided *p*-value < 0.05 was considered statistically significant.

## Results

In total, 167 eyes from 167 DM patients with UCVH met the inclusion criteria during the 31-month study period. These included 103 male and 64 female patients with a mean age of 52.9 years (range 26–81). From these patients, 70 eyes underwent 23G sutureless PPV and 97 eyes underwent 25G sutureless PPV.

The clinical characteristics and operation-related variables of the participants are shown in Table 1. Clinical characteristics, including gender, age, preoperative SBP, preoperative FBSL, serum Cr, urea and pre-PRP agents, did not differ significantly between Groups 23G and 25G. Duration of DM was shorter in Group 23G than in Group 25G (10 years vs 12 years). In particular, PT, APTT, PT-INR, pre- and postoperative IOP in all patients were in a normal range and as such were excluded from subsequent analyses. The two groups differed in operation-related characteristics such as TA usage, electoral-coagulation usage, air-fluid exchange, and anti-VEGF drug usage.

**Table 1:** Clinical characteristics and operation-related variables of the study participants

Characteristics	23G	25G	p-value
Eyes, n	70	97	
Male, n(%)	43 (61.4)	60 (61.9)	0.955
Age, years (mean ± SD)	54.70 ± 11.31	51.63 ± 12.74	0.109
Preoperative systolic blood pressure,mmHg (mean ± SD)	138.01 ± 18.42	136.13 ± 20.17	0.539
Diabetes			
Duration, years	10.00 (5.00–15.00)	12.00 (6.50–16.50)	0.041
Preoperative fasting blood glucose level, mmol/L	5.62 (4.70–7.41)	5.96 (5.05–7.19)	0.639
Serum creatinine, μmol/L	77.55 (58.78–104.93)	76.00 (59.15–127.00)	0.359
Urea, mmol/L	6.51 (5.41–8.88)	6.67 (4.81–8.90)	0.502
Triamcinolone acetonide usage, n(%)	63 (90.0)	59 (60.8)	< 0.001
Elctral-coagulation usage, n(%)	12 (17.1)	7 (7.2)	0.046
Air-fluid exchange, n(%)	66 (94.3)	66 (68.0)	< 0.001
Pre-pan-retinal photocoagulation, n(%)	12 (17.1)	15 (15.5)	0.771
Anti-vascular endothelial growth drug usage, n(%)	10 (14.3)	33 (34.0)	0.004
Intraocular pressure, mmHg			
preoperative IOP	16.45 (15.38–17.53)	16.40 (14.25–17.40)	0.092
postoperative IOP	16.00 (14.93–16.78)	15.31 (14.25–16.40)	0.078

A 6-month follow-up was achieved in all patients from both groups. The incidence and severity of RVH across groups is shown in Table 2. The incidences of postoperative RVH were 25.7 and 21.6% in Groups 23G and 25G, respectively. There were no significant differences between the two groups (*p* = 0.540). There were 3(4.3%) and 5(5.2%) severe postoperative RVH cases (Grade 2) in Groups 23G and 25G, respectively, which ultimately required additional surgeries (e.g., vitreous lavage or PPV). There were no significant differences in postoperative RVH severity between the two groups (*p* = 0.584). Delayed RVH occurred in 6 eyes ((8.6%) in Group 23G and in 8 eyes (8.2%) in Group 25G (*p* = 0.789) (Table 3).

**Table 2:** Incidence rate and severity of RVH

	23G	25G	p-value
Incidence rate			
RVH, n(%)	18 (25.7)	21 (21.6)	0.540
No RVH, n(%)	52 (74.3)	76 (78.4)	
Grade			
Grade 0, n(%)	52 (74.3)	76 (78.35)	0.584
Grade 1, n(%)	15 (21.4)	16 (16.5)	
Grade 2, n(%)	3 (4.3)	5 (5.15)

**Table 3:** Immediate RVH and delayed RVH

	23G	25G	p-value
immediate RVH, n	12	13	0.789
delayed RVH, n	6	8	
no RVH,n	52	76	

The analysis of the individuals with or without postoperative RVH is shown in Table 4. Patient age (*p* = 0.006, *p* < 0.001), duration of DM (*p* = 0.007, *p* = 0.003), and serum Cr (*p* = 0.002, *p* = 0.009) were significantly different between participants with and without postoperative RVH in both Groups 23G and 25G. Additionally, the level of urea (*p* = 0.005) was significantly different between participants with and without postoperative RVH in Group 25G. A binomial logistic regression analysis revealed risk factors of postoperative RVH (Table 5). Age (*p* = 0.019, *p* = 0.004), duration of DM (*p* = 0.046, *p* = 0.02), and serum Cr levels (*p* = 0.006, *p* = 0.042) were significantly different between participants with and without postoperative RVH in both Groups 23G and 25G.

**Table 4:** Characteristics of individuals with or without postoperative RVH

	23G(*n* = 70)			25G(*n* = 97)		
	No RVH(*n* = 52)	RVH(*n* = 18)	p-value	No RVH(*n* = 76)	RVH(*n* = 21)	p-value
Sex (male/female)	31 (59.6)	12 (66.7)	0.596	46 (60.5)	14 (66.7)	0.608
Age, years (mean ± SD)	56.94 ± 10.45	48.22 ± 11.46	0.006	53.96 ± 11.85	43.19 ± 12.52	< 0.001
Duration of diabetes, years	10.00 (5.00–17.00)	5.00 (3.00–9.25)	0.007	12.50 (8.50–17.00)	6.00 (2.00–15.00)	0.003
Preoperative fasting blood glucose level, mmol/L	5.88 (4.88–7.44)	5.27 (3.99–6.97)	0.149	5.93 (5.09–7.06)	6.04 (4.21–7.97)	0.646
Preoperative systolic blood pressure,mmHg	137.60 (100–183)	139.22 (92–187)	0.702	135.03 (88–190)	140.14 (100–187)	0.464
Serum creatinine, μmol/L	70.40 (53.33–88.78)	108.80 (66.78–231.28)	0.002	71.80 (56.08–118.00)	95.00 (68.80–373.60)	0.009
Urea, mmol/L	6.47 (5.17–7.58)	8.14 (5.42–13.36)	0.066	5.88 (4.79–7.81)	9.40 (5.49–14.04)	0.005
Triamcinolone acetonide usage, n(%)	48 (92.3)	15 (83.3)	0.523	45 (59.2)	14 (66.7)	0.536
Electral-coagulation usage (fibrovascular membrane), n(%)	10 (19.2)	2 (11.1)	0.671	6 (7.9)	1 (4.8)	0.988
Air-fluid exchange, n(%)	49 (94.2)	17 (94.4)	1	52 (68.4)	14 (66.7)	0.879
Pre-pan-retinal photocoagulation, n(%)	9 (17.3)	3 (16.7)	1	14 (18.4)	1 (4.8)	0.233
Anti-vascular endothelial growth drug usage, n(%)	9 (17.3)	1 (5.6)	0.402	25 (32.9)	8 (38.1)	0.656

**Table 5:** Results of binomial logistic regression analysis for risk factors of postoperative RVH

	23G				25G			
	Univariate		Multivariate		Univariate		Multivariate	
	OR(95%CI)	*p*-value	OR(95%CI)	p-value	OR(95%CI)	p-value	OR(95%CI)	p-value
Sex (male/female)	1.355 (0.440–4.176)	0.597	4.226 (0.476–37.514)	0.196	0.767 (0.277–2.120)	0.609	2.509 (0.348–18.093)	0.361
Age, years	0.927 (0.877–0.980)	0.008	0.894 (0.814–0.982)	0.019	0.925 (0.881–0.970)	0.001	0.888 (0.819–0.963)	0.004
Duration of diabetes, years	0.872 (0.784–0.971)	0.012	0.833 (0.697–0.997)	0.046	0.888 (0.814–0.968)	0.007	0.835 (0.716–0.972)	0.02
Preoperative fasting blood glucose level	0.784 (0.586–1.048)	0.101	0.928 (0.628–1.371)	0.708	1.068 (0.887–1.287)	0.488	1.182 (0.851–1.640)	0.319
Preoperative systolic blood pressure	1.005 (0.976–1.035)	0.745	1.019 (0.969–1.072)	0.467	1.013 (0.989–1.037)	0.304	0.993 (0.948–1.041)	0.783
Serum creatinine, μmol/L	1.013 (1.002–1.024)	0.02	1.028 (1.008–1.049)	0.006	1.006 (1.002–1.010)	0.004	1.009 (1.000–1.019)	0.042
Blood urea	0.995 (0.972–1.019)	0.703	0.986 (0.929–1.046)	0.637	1.285 (1.107–1.491)	0.001	1.226 (0.968–1.553)	0.09
Triamcinolone acetonide usage	0.417 (0.084–2.075)	0.285	0.626 (0.057–6.861)	0.701	1.378 (0.499–3.806)	0.536	3.777 (0.512–27.84)	0.192
Electral-coagulation usage (fibrovascular membrane)	0.525 (0.104–2.663)	0.437	1.103 (0.057–21.471)	0.948	0.583 (0.066–5.132)	0.627	0.187 (0.006–6.260)	0.349
Air-fluid exchange	1.041(.101–10.692)	0.973	0.156 (0.005–5.348)	0.303	0.923 (0.330–2.581)	0.879	0.858 (0.124–5.934)	0.876
Pre-pan-retinal photocoagulation	0.956 (0.228–4.004)	0.95	2.350 (0.221–25.009)	0.479	0.221 (0.027–1.791)	0.157	0.109 (0.008–1.528)	0.1
Anti-vascular endothelial growth drug usage	0.281 (0.033–2.391)	0.245	0.083 (0.003–2.690)	0.161	1.255 (0.461–3.420)	0.657	0.456 (0.074–2.795)	0.396

## Discussion

With the rapid development of surgical techniques and instruments, the incidence of postoperative RVH in PDR has fallen significantly from 75% in the 1980s to approximately 11.8–40% [11, 12, 15, 16]. Khuthaila et al. reported an incidence rate of 32% for postoperative RVH with 23G PPV in PDR patients [17], while Mahallngam et al. reported an RVH incidence of 21.6% [18]. Similarly, in the present study, the incidences of postoperative RVH were 25.7 and 21.6% in Groups 23G and 25G, respectively.

Research is ongoing to elucidate the risk factors of postoperative RVH. Mahallngam et al. and Tolentino et al. reported that a younger patient age was significantly associated with postoperative RVH [18, 19]. In the present study also, the age influenced postoperative RVH. There are two possible explanations for this; one related to surgery and the other with patients themselves. First, it is more difficult to induce a complete posterior vitreous detachment in younger patients than the older ones because of their stronger vitreoretinal adhesions. When a residual split posterior vitreous cortex remains firmly attached to the retina, it serves as a natural scaffold for the proliferation of neovascularisation membrane. Vitreous contraction and subsequent traction on unhealthy fibrovascular membranes can induce postoperative re-bleeding. A second explanation for the impact of patient age on postoperative RVH is that the onset of PDR in younger patients indicates a more rapid disease progression and thus, more aggressive disease. In some cases, this also indicates a broader area of active neovascularisation. Given this, the increasing numbers of unhealthy neovascular vessels bleed easily.

The present study also identified the duration of DM as a predictor of increased postoperative RVH risk. Epidemiologic data reveals that PDR almost never develops within the first 10 years of DM onset [20]. But in our study, the patients happened postoperative RVH experienced less than 10 years duration (5 years, 23G VS 6 years, 25G). The estimated prevalence of diabetes in China is 12%, or over 100 million patients [21, 22]. The burden of diabetes has consistently risen in China over the past two decades and is predicted to continue to rise [21, 22] and most of these cases are undiagnosed [21, 22]. Combined with the current situation of diabetes in China, a possible explanation for this is that some patients lack an awareness of their DM and their disease may develop for many years without any diagnosis or treatment. The shorter duration provided by the patient is not the real duration of DM. We hypothesized that a shorter duration of DM at the time of presentation may reflect the patient’s ignorance of the disease, leaving it uncontrolled for many years. In patients with a longer duration of DM, treatment might have been used more consistently and for a longer period; therefore, their degree of PDR may be less severe. More studies should be made to further study the relationship between the shorter duration of DM at the time of presentation and the postoperative RVH. Therefore, we contend that the clinicians should pay more attention to the patients with a shorter DM onset time (and thus later detection).

In the present study, we reported for the first time that Cr was a novel RVH risk predictor and strongly associated with postoperative RVH. Cr is the most common index used for renal function. Compensatory kidney function is very powerful. The Cr values won’t be increased, unless the degree of kidney damage accounts for more than half of the kidney. Higher Cr values in the present study represented poor renal function and a poor general state of health due to poor control of pre-operation blood sugar levels. Kussman et al. reported that in the typical clinical course of diabetic nephropathy, the mean duration of DM at the onset of early renal function failure was approximately 19 years [23]. So the higher the Cr value reflects the longer duration of DM in reality. Combined with the risk predictor of postoperative RVH mentioned above, a shorter duration of DM at the time of presentation, which confirms again that the disease had been ignored and uncontrolled for many years. Given this, the patients with higher Cr values in the present study may have had more aggressive PDR, thus increasing their risk of postoperative RVH.

As has previously been found, silicone oil and inert gases such as SF6 and C3F8 may decrease the incidence of postoperative RVH, especially in early RVH cases [24]. One possible reason for this may be that longstanding mechanical tamponade of fragile retinal vessels by oil or gas bubbles that occurs with this procedure. This tamponade also concentrates coagulation factors within the close proximity of bleeding sites, thereby promoting the re-establishment of vascular integrity. We, therefore, excluded cases from the present study in which silicone oil or inert gas tamponade appeared to minimise any interference by these factors and to isolate other predictors of postoperative RVH risk.

After the first commercially available narrow-gauge vitrectomy system was described by Fujii et al. in 2002, a high number of narrow-gauge systems were employed worldwide [25]. These revolutionary advances led to faster postoperative recovery, less postoperative discomfort, reduced surgically-induced astigmatisms, and a quicker entry and exit from the eye [26]. Furthermore, 23G devices retained the fluid dynamics and instrumental rigidity offered by 20G systems and required minimal changes in the surgical technique. The 25G system further improved fluidics and rigidity. Owing to these advantages, both two narrow-gauge systems are often used by the surgeons. Thus, in the present study, patients with DR who underwent both sutureless 23G and 25G PPV for NCVH were included. While the incisions created during 25G PPV do not require sutures, 23G PPV incisions sometimes do. As such, we only included the patients who underwent sutureless 23G PPV in the present study to eliminate any influence associated with the sutures.

One of the disadvantages of the sutureless technique is hypotony. It remains unclear whether hypotony is significantly correlated with postoperative RVH; however, both the advantages and disadvantages have been reported previously in the literature [12, 27]. In the present study, both pre- and postoperative IOP were within the normal range. Thus, any relationship between the hypotony and postoperative RVH was not assessed in this study.

The present study has a few limitations. One was the number of patients who used anti-VEGF drugs. Anti-VEGF drug injection before vitrectomy causes regression of active neovascularization, at least in the short term [28]. It reduces the likelihood of intraoperative hemorrhage in diabetic vitrectomy, makes the surgery quicker and easier [29]. However, in the previous literature, it still remains unclear whether anti-VEGF is significantly correlated with postoperative RVH [30]. Due to the high cost of anti-VEGF drugs, few people can afford this treatment and thus, a few patients included in this study used them. Therefore, in the present study, the relationship between anti-VEGF drug use and postoperative RVH remains unclear. A larger sample size is needed to explore this aspect more closely. Another limitation was that there were few cases of delayed or severe postoperative RVH among the participants of the present study. Hence, we were unable to investigate the risk factors associated with delayed and severe postoperative RVH. Third, only preoperative FBSL were recorded. A high level of preoperative FBS only reflected the difficulty of glycemic control during a short period, and can be influenced by many facts including perioperative stress. Further studies are required to clarify the relationship between perioperative hyperglycemia and postoperative complications. Fourth, due to the small number of patients who implemented PRP before operation, the relationship between preoperative PRP and postoperative RVH could not be observed in our study. Because of the huge number of Chinese diabetes patients, few patients can follow the doctor’s instructions to regularly check the blood glucose level and eye conditions, a lot of patients fail to implement PRP in time, but directly have VH, and have to do surgical intervention. Fifth, number of patients with neovascularization membrane is small, no positive results can be observed in our study, but this does not prove that there is no correlation between neovascularization membrane and postoperative RVH. In the future study, we are going to collect more patients for further in-depth and sub analysis.

## Conclusions

The incidence and severity of RVH were 25.7 and 4.3%, respectively, in Group 23G and 21.6 and 5.2%, respectively, in Group 25G. The findings of our study suggest that 23G sutureless vitrectomy was as safe as 25G sutureless vitrectomy for NCVH in patients with DR. The practical applications of the study to clinical domains and public policy deserved attention. A younger age, shorter duration of DM, and higher Cr levels were significant predictors of postoperative RVH risk in patients who underwent sutureless vitrectomy.

## Data Availability

The datasets used and analyzed during the current study are available from the corresponding author on reasonable request.

## Abbreviations

23G or 25G: 23- or 25-gauge narrow-gauge
APTT: Activated partial thromboplastin time
Cr: Creatinine
DM: Diabetes mellitus
DR: Diabetic retinopathy
FBSL: Fasting blood sugar level
IOP: Intraocular pressure
NCVH: Non-clearing vitreous haemorrhage
PDR: Proliferative diabetic retinopathy
PPV: Pars plana vitrectomy
PRP: Pan-retinal photocoagulation
PT: Prothrombin time
PT-INR: Prothrombin standardisation ratio
RVH: Recurrent vitreous haemorrhage
SBP: Systolic blood pressure
TA: Triamcinolone acetonide
VEGF: Vascular endothelial growth factor
VH: Vitreous haemorrhage

## References

[1] Rathmann W, Giani G (2004). Global prevalence of diabetes: estimates for the year 2000 and projections for 2030. Diabetes Care.

[2] Whiting DR, Guariguata L, Weil C, Shaw J (2011). IDF diabetes atlas: global estimates of the prevalence of diabetes for 2011 and 2030. Diabetes Res Clin Pract.

[3] Fong DS, Aiello L, Gardner TW, King GL, Blankenship G, Cavallerano JD (2004). Retinopathy in diabetes. Diabetes Care.

[4] Elman MJ, Ayala A, Bressler NM, Browning D, Flaxel CJ, Glassman AR (2015). Intravitreal ranibizumab for diabetic macular edema with prompt versus deferred laser treatment: 5-year randomized trial results. Ophthalmology..

[5] Nguyen QD, Brown DM, Marcus DM, Boyer DS, Patel S, Feiner L (2012). Ranibizumab for diabetic macular edema: results from 2 phase III randomized trials: RISE and RIDE. Ophthalmology..

[6] Gross JG, Glassman AR, Jampol LM, Inusah S, Aiello LP, Antoszyk AN (2015). Panretinal photocoagulation vs intravitreous ranibizumab for proliferative diabetic retinopathy: a randomized clinical trial. JAMA..

[7] Bhavsar AR, Torres K, Glassman AR, Jampol LM, Kinyoun JL (2014). Evaluation of results 1 year following short-term use of ranibizumab for vitreous hemorrhage due to proliferative diabetic retinopathy. JAMA Ophthalmol.

[8] Shi L, Yi-Fei H (2012). Postvitrectomy diabetic vitreous hemorrhage in proliferative diabetic retinopathy. J Res Med Sci.

[9] Yorston D, Wickham L, Benson S, Bunce C, Sheard R, Charteris D (2008). Predictive clinical features and outcomes of vitrectomy for proliferative diabetic retinopathy. Br J Ophthalmol.

[10] Yang CM (1998). Surgical treatment for diabetic retinopathy: 5-year experience. J Formos Med Assoc.

[11] Schachat AP, Oyakawa RT, Michels RG, Rice TA (1983). Complications of vitreous surgery for diabetic retinopathy: II. Postoperative complications Ophthalmology.

[12] Lee BJ, Yu HG (2010). Vitreous hemorrhage after the 25-gauge transconjunctival sutureless vitrectomy for proliferative diabetic retinopathy. Retina..

[13] Yan H, Cui J, Lu Y, Yu J, Chen S, Xu Y (2010). Reasons for and management of postvitrectomy vitreous hemorrhage in proliferative diabetic retinopathy. Curr Eye Res.

[14] Zaninetti M, Petropoulos IK, Pournaras CJ (2005). Proliferative diabetic retinopathy: Vitreo-retinal complications are often related to insufficient retinal photocoagulation. J Fr Ophtalmol.

[15] Fujii GY, De Juan E, Humayun MS, Chang TS, Pieramici DJ, Barnes A (2002). Initial experience using the transconjunctival sutureless vitrectomy system for vitreoretinal surgery. Ophthalmology.

[16] Sima P, Zoran T (1994). Long-term results of vitreous surgery for proliferative diabetic retinopathy. Doc Ophthalmol.

[17] Yeh PT, Yang CM, Yang CH, Huang JS (2005). Cryotherapy of the anterior retina and sclerotomy sites in diabetic vitrectomy to prevent recurrent vitreous hemorrhage: an ultrasound biomicroscopy study. Ophthalmology.

[18] Khuthaila MK, Hsu J, Chiang A, DeCroos FC, Milder EA, Setlur V (2013). Postoperative vitreous hemorrhage after diabetic 23-gauge pars plana vitrectomy. Am J Ophthalmol.

[19] Tolentino FI, Cajita VN, Gancayco T, Skates S (1989). Vitreous hemorrhage after closed vitrectomy for proliferative diabetic retinopathy. Ophthalmology.

[20] Klein R, Klein BE, Moss SE, Davis MD, DeMets DL (1984). The Wisconsin epidemiologic study of diabetic retiopathy. II. Prevalence and risk of diabetic retinopathy when age at diagnosis is less than 30 years. Arch Ophthalmol.

[21] Xu Y, Wang L, He J, Bi Y, Li M, Wang T (2013). Prevalence and control of diabetes in Chinese adults. Jama..

[22] Yang W, Lu J, Weng J, Jia W, Ji L, Xiao J (2010). Prevalence of diabetes among men and women in China. N Engl J Med.

[23] Kussman MJ, Goldstein H, Gleason RE (1976). The clinical course of diabetic nephropathy. JAMA.

[24] Mahalingam P, Topiwalla TT, Ganesan G (2018). Vitreous rebleed following sutureless vitrectomy: incidence and risk factors. Indian J Ophthalmol.

[25] Yang CM, Yeh PT, Yang CH (2007). Intravitreal long-acting gas in the prevention of early postoperative vitreous hemorrhage in diabetic vitrectomy. Ophthalmology.

[26] Nagpal M, Wartikar S, Nagpal K (2009). Comparison of clinical outcomes and wound dynamics of sclerotomy ports of 20, 25, and 23 gauge vitrectomy. Retina.

[27] Altan T, Acar N, Kapran Z, Unver YB, Ozdogan S (2008). Transconjunctival 25-gauge sutureless vitrectomy and silicone oil injection in diabetic tractional retinal detachment. Retina.

[28] Yang CM, Yeh PT, Yang CH, Chen MS (2008). Bevacizumab pretreatment and long-acting gas infusion on vitreous clear-up after diabetic vitrectomy. Am J Ophthalmol.

[29] Rizzo S, Genovesi-Ebert F, Di Bartolo E, Vento A, Miniaci S, Williams G (2008). Injection of intravitreal bevacizumab (Avastin) as a preoperative adjunct before vitrectomy surgery in the treatment of severe proliferative diabetic retinopathy (PDR). Graefes Arch Clin Exp Ophthalmol.

[30] Berk Ergun S, Toklu Y, Cakmak HB, Raza S, Simsek S (2015). The effect of intravitreal bevacizumab as a pretreatment of vitrectomy for diabetic vitreous hemorrhage on recurrent hemorrhage. Semin Ophthalmol.

